# Heterogeneity of Autism Characteristics in Genetic Syndromes: Key Considerations for Assessment and Support

**DOI:** 10.1007/s40474-023-00276-6

**Published:** 2023-05-09

**Authors:** Lauren Jenner, Caroline Richards, Rachel Howard, Joanna Moss

**Affiliations:** 1 School of Psychology, University of Surrey, Guildford, England; 2 School of Psychology, University of Birmingham, Birmingham, UK

**Keywords:** Autism, Genetic syndromes, Intellectual disability, Heterogeneity, Co-occurrence, Behavioural phenotypes

## Abstract

**Purpose of Review:**

Elevated prevalence of autism characteristics is reported in genetic syndromes associated with intellectual disability. This review summarises recent evidence on the behavioural heterogeneity of autism in the following syndromes: Fragile X, Cornelia de Lange, Williams, Prader-Willi, Angelman, Down, Smith-Magenis, and tuberous sclerosis complex. Key considerations for assessment and support are discussed.

**Recent Findings:**

The profile and developmental trajectory of autism-related behaviour in these syndromes indicate some degree of syndrome specificity which may interact with broader behavioural phenotypes (e.g. hypersociability), intellectual disability, and mental health (e.g. anxiety). Genetic subtype and co-occurring epilepsy within syndromes contribute to increased significance of autism characteristics. Autism-related strengths and challenges are likely to be overlooked or misunderstood using existing screening/diagnostic tools and criteria, which lack sensitivity and specificity within these populations.

**Summary:**

Autism characteristics are highly heterogeneous across genetic syndromes and often distinguishable from non-syndromic autism. Autism diagnostic assessment practices in this population should be tailored to specific syndromes. Service provisions must begin to prioritise needs-led support.

## Introduction

People with genetic syndromes associated with intellectual disability (ID) are more likely to evidence clinically significant autism characteristics compared to people in the general population [1]. For many people with genetic syndromes associated with ID, characteristics related to the ‘core’ diagnostic criteria of autism are evident [2••]. However, detailed analyses consistently indicate that the profile and developmental trajectory of autism characteristics across these groups are highly heterogeneous, in ways which indicate some degree of syndrome specificity (e.g. in Fragile X syndrome; 3). Furthermore, there is a tendency for people within these populations to demonstrate profiles of autism characteristics that are phenotypically distinct, in subtle and specific ways, from that of non-syndromic^1^ autism [4]. The picture is further complicated by the fact that ID is a primary characteristic of these genetic syndromes. The extent to which associated ID contributes to the heterogeneity of autism characteristics in people with genetic syndromes has not been clearly established and is likely to be variable across syndrome groups [1]. Furthermore, differential diagnosis, particularly in those with severe to profound ID, is challenging [5••], both conceptually and practically. Together, these complexities confer significant challenges for assessment and diagnosis of autism in people with genetic syndromes and likely explain the substantially reduced and delayed recognition of autism in clinical practice for these individuals and their families [6, 7••]. Further delineation of these factors within and between genetic syndrome groups, alongside greater precision of assessment of autism for this population as whole, will be critical to address the extant gap between research reported rates of autism characteristics and the interpretation and application of these findings within clinical practice.

In this paper, we first review recent work on autism in genetic syndromes associated with ID, with an emphasis on raising awareness and understanding of phenotypic heterogeneity of autism characteristics within specific genetic syndromes. We then highlight the need to advance developments in diagnostic assessment tools and autism-related support for people with genetic syndromes.

## Prevalence of Autism in Genetic Syndromes

Research over recent years has indicated significantly elevated rates of autism and related characteristics in several genetic syndromes associated with ID [1, 2••]. Prevalence estimates within the general population indicate rates of autism of at least 1% [8]. However, people with a genetic syndrome associated with ID are reported to be at least ten times more likely to show autism characteristics than the general population [1]. Yet application of these findings in clinical diagnostic services is somewhat limited [6], with reports of significantly delayed and reduced access to autism diagnostic pathways in these populations. For example, the age of autism diagnosis in individuals with Sturge-Weber syndrome is over the age of 8 years old, with 94% of individuals being diagnosed in a Tier 3 specialist service [7••].

In some cases, this research has challenged existing stereotypes of particular syndrome groups. For instance, individuals with Down syndrome have historically been characterised as having social communication skills that directly contrast with the diagnostic characteristics of autism [9]. However, reported prevalence rates of autism in Down syndrome have increased from 5 to 42% in the past 20 years [10, 11]. A similar trend has also been documented in Williams syndrome [12]. The apparent increase in the reported prevalence of autism within these populations may reflect an improved understanding and awareness of the co-occurrence of autism in genetic syndromes. However, these and other prevalence data continue to be drawn from an application of cut-off scores from diagnostic autism measures, which have been developed and normed in the general population, where there will be limited representation of syndromic autism. As such, these prevalence data assume a similar constellation of autism characteristics that contribute to scoring at diagnostic threshold to that seen in non-syndromic autism; this assumption may mask important syndrome-specific profiles of autism characteristics.

## Heterogeneous Profiles of Characteristics

Autism is widely understood as a complex condition, with variation in terms of sex-specific factors, intellectual ability, and co-occurring conditions [13]. Detailed analyses that have considered the specific patterns of autism characteristics within individual syndrome groups show that the profiles of autism characteristics are highly heterogeneous between different syndrome groups [14••], even when individuals score above clinical cut-off scores on autism assessment tools. In many cases, the profile of autism characteristics is reported to be subtly different, both qualitatively and quantitatively from non-syndromic autism. For example, some syndrome groups evidence a profile of characteristics which includes significant repetitive behaviours and/or interests (RRBIs) alongside differences in social communication that are similar to that of autistic people who do not have a genetic syndrome, combined with comparatively heightened social motivation (e.g. Rubinstein-Taybi syndrome [14••], Sturge-Weber syndrome [7••]). For other syndromes, both social interaction and communication differences evidence similarities with non-syndromic autism, while RRBIs may be less apparent in the syndrome or may present differently to those described in autistic people without a syndrome (e.g., Phelan-McDermid syndrome [15], Sotos [16]). In Fragile X syndrome, which has been understood to be the leading monogenic cause of autism over the past 30 years [18], a deep phenotyping approach is now commonly adopted, leading researchers to argue that the profile of autism characteristics present are not captured fully by categorical diagnosis alone [19–21, 22•]. Such variation is not necessarily indicative of reduced presence of autism characteristics in these populations. Rather, they suggest that there are unique autism-related strengths and challenges in people with genetic syndromes. These may differ from that of non-syndromic autism and may differ between syndromes. These similarities and differences need to be recognised to ensure that people receive the most appropriate support.

## Developmental Trajectories and Changes with Age

In the general population, subgroup differences (e.g., sex-specific) in the longitudinal heterogeneity of autism characteristics reveal fractionable trajectories which are not clearly related to the development of language and functioning [23]. Longitudinal heterogeneity of autism characteristics is also variable across and within different genetic syndromes. In Cornelia de Lange syndrome, autism characteristics are reported to become more evident with age, specifically in relation to social interaction skills [24••]. A similar increase in autism characteristics from childhood to adulthood has also been reported in Sotos syndrome [25]. However, there is a question surrounding whether these differences are attributable to age or changes in context. For instance, in Sotos syndrome, autism characteristics were reported to increase during the COVID-19 pandemic, a change not seen in age- and IQ-matched autistic children [26]. It can also be difficult to distinguish an increase in autism characteristics from other changes which commonly co-occur with age in syndromes, particularly the emergence of mental health conditions such as psychosis (e.g., 22q11.2 deletion syndrome, [26], Prader-Willi syndrome [27]) and anxiety (e.g., Fragile X syndrome; 20). Improved understanding of these trajectories and interacting factors is critical to ensure people receive the timeliest support and provide clarity in diagnostic classifications at all ages.

## Broader Phenotypic Characteristics

One explanation for the so-called atypical profiles of autism in genetic syndromes might be that broader phenotypic behaviours associated with a given syndrome interact with the profile of autism characteristics, resulting in a syndrome-specific signature of autism-related strengths and challenges which vary across the lifespan. Disentangling autism characteristics from the broader phenotypic characteristics of the syndrome is therefore incredibly complex. There are several implications of such heterogeneity within clinical practice. First, the presence of the syndrome may lead to diagnostic overshadowing [6], resulting in delayed assessment and diagnosis [7••]. Second, an autism diagnosis is given, but without clear understanding of the unique profile of strengths and challenges for that person—information which should guide more tailored support. Finally, although perhaps less likely, individuals with rare syndromes may meet diagnostic threshold for autism as a result of concomitant syndrome- or ID -related characteristics, and the autism diagnosis may in fact be less appropriate.

In the following sections, we outline recent evidence which highlights the key considerations for understanding the heterogeneity of autism characteristics in genetic syndromes. As it is beyond the scope of this paper to review all syndromes systematically, we have selected eight syndromes which have relatively large bodies of empirical evidence in relation to autism characteristics. Each provides examples of key considerations for clinicians and researchers seeking to understand autism in these populations. The presentation of autism characteristics in these syndromes should be interpreted within the context of the broader behavioural phenotype associated with each genetic syndrome, summarised in Table 1.

**Table 1:** Overview of the phenotypic profile and key considerations for understanding autism in genetic syndromes

	**Genetic cause**	**Degree of intellectual disability (ID)**	**Broader behavioural phenotype**	**Autism-related considerations**
Fragile X syndrome (FXS)	Expanded CGG repeat sequence (> 200 repeats) on the FMR1 gene located at Xq27.3 [121]	Males tend to have **moderate** to **severe** degree of ID. Females tend to be less clearly affected, presenting with a more variable profile of ability [121]	**Socially avoidant** behaviours (e.g., **gaze aversion**) are highly characteristic [21, 31, 32]. Difficulties with sustained **attention**, **impulsiveness**, and **overactivity** are reported [40, 41]. Frequent attempts to escape interactions and demands [49]. High co-occurrence of **ADHD** and **anxiety**, particularly **social phobia** [22•]. **Sensory sensitivities** are also common [122]	• Reduced eye-contact [21, 31] and social avoidance [32] • Social smiling and complex mannerisms [36] • Repetitive speech and stereotyped behaviours [36] • ID severity [33], anxiety, ADHD [40, 41], and epilepsy [29]
Cornelia de Lange syndrome (CdLS)	Mutation in one of seven genes: NIPBL, SMC1A, SMC3, RAD21, BRD4, HDAC8, and ANKRD11. Mosaicism is reported in approximately 23% of cases [123]	Degree of ID severity is wide, but most people fall within the **moderate** ID range [123]	Common characteristics include **selective mutism**, **extreme shyness**, **social anxiety**, and **social avoidance** [49]. Social anxiety, **low mood**, **impulsivity**, and **insistence on sameness** increases with age [51–53]. Health issues (e.g., gastroesophageal reflux) are prominent and associated with **self-injury** and low mood [123]	• Social anxiety [44–46] and intolerance of uncertainty [47] • Familiarity and social context [49] • Age-related increase in social withdrawal, self-injury and low mood [51–53]
Williams syndrome (WS)	Microdeletion of 26–28 genes on chromosome 7q11.23 [124]	Typically, degree of ID severity is in the **borderline** to **moderate** range [58]	High motivation for social engagement, presenting as **overly friendly** and **affectionate **[55]. Heightened approach towards strangers [61]. Relative strengths in **expressive language** [125]. **Visuo-spatial processing** difficulties and **auditory hypersensitivity **[56, 123]. **Obsessive preoccupations** [57] and increased **anxiety** are reported [58, 59]	• Hypersociability [61] and social vulnerability [62] • Auditory hypersensitivity [56] • Repetitive behaviours [57] • Intolerance of uncertainty [60•] • Age-related increase in anxiety [58, 59]
Prader-Willi syndrome (PWS)	Loss of the paternally expressed genes on the q11–13 region of chromosome 15, due to deletion or inheritance of two maternal copies [73]	**Mild** to **moderate** degree of ID is common**.** Greater cognitive difficulties are associated with the UPD subtype than the deletion [73]	**Hyperphagia** can cause excessive eating and food stealing, which requires strict food management [73]. Frequent and severe **temper outbursts** are also common [66]. Other features include **hoarding**, **repetitive questioning**, preference for **routine/insistence on sameness** and repetitive **skin-picking **[63]. **Psychiatric conditions** also frequently co-occur [68]	• Difficulty with overall rapport, social response, and social overtures [69] • Reduced eye contact and emotional expressions (69) • Insistence on sameness, strong interests, repetitive questioning, and compulsivity [63] • ID severity and genetic subtype [73] • Psychiatric conditions [68]
Angelman syndrome (AS)	Loss of the maternal copy of the UBE3A gene on chromosome 15, due to deletion or inheritance of two paternal copies [127]	Degree of ID severity is often **severe** to **profound**. A larger deletion has been associated with greater cognitive difficulties [77]	Strong use of **non-verbal communication** (e.g. gesture, signs, and other systems) [75]. High levels of **smiling** and **laughing** are seen in response to adult attention [69, 74]. **Separation distress** can lead to **aggressive behaviours**, such as hair pulling and grabbing people [128]. Other features include **sensory processing differences**, **hyperactivity**, **inattention**, and **sleep difficulties** [129]. **Seizures** are common [78]	• Shared enjoyment of interaction [69] • Increased eye contact, smiling, laughing [74] and gesture [75] • Age-related decline in social motivation [74] • Genetic subtype and epilepsy [78]
Down syndrome (DS)	Trisomy of chromosome 21 [130]	ID severity ranges from **mild** to **severe** [79]	Relative strengths in **social functioning** [85]. Challenges with **language production** and **verbal short-term memory**. Preference for **visual communication** systems [131]. Strong use of **gesture** and **body language**. Behavioural difficulties include **impulsivity**, **rigidity**, and **oppositional behaviour **[83]. Accelerated aging and increased likelihood of developing **Alzheimer’s** disease [132]	• Strengths in social smiling, eye contact, offering comfort, social overtures [80] • ID severity [9, 79] and language delays [83] • Behaviours that challenge [9, 83, 86]
Smith-Magenis syndrome (SMS)	A deletion or mutation of the RAI1 gene on chromosome 17 [88]	A **moderate** degree of ID is common [88]	**Sleep problems** are highly characteristic, due to an inverted synthesis of melatonin [133]. A strong desire for **adult attention** and **attachment to particular people** is reported [89]. Other characteristic difficulties include **self-injury**, **aggressive behaviours**, and elevated **impulsivity** [88]. There are also **stereotyped behaviours **[90], such as ‘self-hugging’, repetitive page turning, and teeth grinding	• Strong social motivation but more negative outcomes [89] • Behavioural and emotional difficulties decrease with age [90] • Reversed sex difference (female > males) [91]
Tuberous sclerosis complex (TSC)	Mutation in the TSC1 gene on chromosome 9 or TSC2 gene on chromosome 16 [134]	Ranges from **average IQ** for the general population to **profound ID**. Earlier onset and greater severity of **seizures** is related to more severe ID [104]	**Seizures** are one of the most common (> 80%) features [93•]. Other characteristic difficulties include **social withdrawal**, **aggression**, **self-injury**, and **mood swings**. High rates of **anxiety** and **depression** are reported [135]. **ADHD** also frequently co-occurs [136]	• Social communication differences more evident than RRBIs in infancy [101, 102] • Seizure onset, frequency, and language development [103]

Key terms in bold for ease of interpretation

### Fragile X Syndrome (FXS)

In FXS, half of males and nearly 20% of females meet DSM-5 criteria for autism spectrum disorder^2^ (ASD; 29). Social anxiety is characteristic of FXS and overlaps behaviourally with autism characteristics [21], together impacting day-to-day functioning [30]. Studies have indicated that autism is a distinct condition in FXS that can be dissociated from the broader behavioural phenotype. From infancy, differences in reactions to strangers [31], social avoidance [32], reduced eye contact [21, 31], behavioural inflexibility [33], and behaviours that challenge [29], distinguish individuals with FXS who score above threshold for autism. In fact, more similarities are seen between those with non-syndromic autism and FXS (+ autism^3^) than between those with FXS (+ autism) and FXS-alone [33]. However, reliance on current diagnostic algorithms masks heterogeneity inherent to the behavioural phenotype. For instance, young males with FXS who score above threshold on the Autism Diagnostic Inventory-Revised (ADI-R; 34) present with qualitatively different characteristics than age-matched males with non-syndromic autism, such as increased social smiling and complex mannerisms [36]. On the Autism Diagnostic Observational Schedule (ADOS; 39), increased repetitive speech, stereotyped behaviours, and hyperarousal are reported to distinguish those with FXS from non-syndromic autism [36]. These findings highlight the need to look beyond prescriptive algorithms, even when the behaviour presented appears distinct within the syndrome, and similar to non-syndromic autism. Furthermore, the onset of autism characteristics and their developmental trajectory in males with FXS differs relative to males with non-syndromic autism [3] due to differences in cognitive ability and expressive language [38]. Even within FXS, differences in the developmental trajectory of autism characteristics have been evidenced related to impulsivity [39], the presence of co-occurring attention deficit hyperactivity disorder (ADHD) [40, 41], adaptive functioning [33], and epilepsy [29]. It is therefore important that the time course of autism characteristics is understood within the context of co-occurring conditions and support addresses these simultaneously across development.

### Cornelia de Lange Syndrome (CdLS)

Up to 45% of people with CdLS meet diagnostic cut-off for autism on the ADOS [42]. Autism characteristics are more prominent among those with greater severity of physical phenotypic features in CdLS (e.g., upper limb differences; 42). Social anxiety distinguishes those with CdLS from non-syndromic autism [44, 45] and is positively associated with the prevalence of autism characteristics across the lifespan, independent of IQ [46]. Intolerance of uncertainty appears to mediate the relationship between autism characteristics and anxiety in CdLS [47], as described in non-syndromic autism [48]. During interactions with an unfamiliar adult and when participation is voluntary, individuals with CdLS show heightened social anxiety and lower social motivation, a finding not evidenced in fragile X, Rubinstein Taybi or Down syndromes [49]. The interplay between social anxiety, autism characteristics, and social context has important implications for the suitability and validity of direct assessment in CdLS (e.g., ADOS-2; 47) and highlights the need for greater precision of assessment in these populations. Age-related differences are also evident, with repetitive behaviours and social withdrawal becoming more prominent among older individuals and increasing over time [23, 40]. Notably, several studies have also associated older age with more frequent self-injury and compulsive behaviour, and lower levels of interest and pleasure in CdLS, indicating that additional challenges are coinciding with age-related changes in autism characteristics in this group [51–53]. The significance of age-related changes experienced by people with CdLS highlights the need to provide additional and/or bespoke support, particularly during the transition to adulthood which is a critical period of change.

### Williams Syndrome (WS)

Using the ADOS, estimates of autism in WS range from 30 to 35%, although some behaviours may be better characterised as part of WS, for example, difficulties with imagination/creativity, gesture, and repetitive behaviours, rather than indicative of an additional autism diagnosis [54]. Hypersociability is considered to be central to the WS phenotype [55], alongside auditory hypersensitivity [56] and repetitive behaviours [57]. People with WS also experience significant anxiety, which increases with age and results in lower social motivation [58, 59]. Similarly to autistic individuals and those with CdLS, intolerance of uncertainty mediates the relationship between anxiety and autism characteristics in WS [60•]. However, unlike CdLS, social interactions are not influenced by degree of familiarity with a partner in WS [61] resulting in increased social vulnerability [62]. These cross-syndrome comparisons indicate subtle differentiations in the presentation of autism characteristics, which arise from phenotypic differences that are key to consider when delivering support. For example, both groups may benefit from support designed for autistic people which improves tolerance of uncertainty to alleviate heightened anxiety (e.g., Coping with Uncertainty in Everyday Situations [CUES©], 61) but people with WS may additionally benefit from supports to mitigate social vulnerability whilst preserving independence.

### Prader-Willi Syndrome (PWS)

In PWS, estimates of autism using the Social Communication Questionnaire (SCQ; 62) can be as high as 29–49%, but when assessed directly by PWS-experts using the ADOS-2, the rate of ASD diagnosis reduces to 12.3% (14 out of 146 children; 63). In this study, people with PWS (+ autism) showed more difficulty with overall rapport and reduced quality in response and overtures compared to those with PWS alone. Insistence on sameness in routines/events and compulsivity were seen in 76–100% of this sample (± autism), appearing related to physiological challenges, including hyperphagia and emotional regulation [66]. Strong interests described as ‘intense obsessionality’ are more marked in PWS than non-syndromic autism [67]. Psychiatric conditions also frequently co-occur, the most common being anxiety, expressed through difficulties with transitions, skin picking, and repetitive questioning [68]. Evidence from PWS demonstrates that syndrome-specific expertise is vital to ensure valid and efficient differential diagnosis. People with PWS also present with social communication differences, such as reduced eye contact, limited range of emotional expressions, and differing quality of social overtures [69]. Social-cognitive differences in PWS are also reminiscent of those described in autistic individuals [70]. The nature of social differences can be distinguished between genetic subtypes within PWS, from as young as 3 years old [71]. When compared to people with deletion subtypes, those with the uniparental disomy or imprinting defect have greater social communication differences and are more likely to be diagnosed with autism [72]—associated with greater severity of ID [73]. The apparent fractionation of social communication difficulties from RRBIs within PWS related to genetic subtype speaks to the differing pathways to behavioural autism characteristics. Findings also indicate that differing clinical support may be warranted for different genotypes *within* syndromes.

### Angelman Syndrome (AS)

AS has been considered to be the ‘sister’ syndrome to PWS, with markedly contrasting phenotypes. People with AS present with fewer autism characteristics compared to those with PWS, particularly within the domain of social affect, such as increased shared enjoyment of social interactions [69]. This difference could be attributed to the strong motivation for social contact seen in AS, characterised by behavioural signatures such as frequent smiling and laughing [74]. Despite people with AS having few or no words, they demonstrate relative strengths in non-verbal communication, particularly through use of gesture and symbols [75]. Children with the non-deletion subtypes often have strengths in these communicative abilities and are more responsive to social reinforcement (e.g., eye contact, laughing [74]) than those with a deletion. Correspondingly, autism characteristics are observed more commonly in deletion subtypes (75%) than uniparental disomy or mutations (11%) using the ADOS-2 [77]. Epilepsy in AS is thought to contribute to the development of autism characteristics to a greater degree than expected from the underlying genetic subtype alone, as those with AS and epilepsy score significantly higher on the SCQ than those without epilepsy [78]. Age-related decline in social motivation [74] and the onset of autism characteristics should be explored further in relation to epilepsy.

### Down Syndrome (DS)

A ‘friendly stereotype’—that individuals are overly sociable—is also associated with DS. The prevalence of co-occurring autism in DS is estimated to be 16–41% [11, 79]. Though some relative strengths in reciprocal social interaction (e.g., social smiling, offering comfort, social overtures) are reported among those with DS who meet screening criteria for autism relative to those with non-syndromic autism [80], broad composite scores are similar across social and non-social diagnostic domains [81, 82]. The majority of people with DS who score highly on the SCQ [9] and ADOS-2 [79] have more severe ID than individuals with DS alone. Specifically, children with DS and clinically significant autism characteristics are more likely to acquire language later and be less likely to communicate using phrases and sentences than children with DS alone [83]. Notably, there is evidence of subgroups of people with DS and severe ID who are not autistic [84], suggesting that the presentation of autism in DS cannot solely be accounted for severity of ID. It has been hypothesised that individuals with DS overcome functional difficulties by adapting to social environments [85]. This may explain why co-occurring DS and autism is associated with greater manifestation of behaviours that challenge relative to non-syndromic autism [86] and DS alone [9, 83]. Recognising autism characteristics in DS could be useful for prioritising and tailoring particular support (e.g., Speech and Language Therapy [86]).

### Smith-Magenis Syndrome (SMS)

The behavioural phenotype of SMS includes sleep disturbances, self-injurious and maladaptive behaviours, stereotypies, and sensory difficulties [88]. When compared to those with DS, children with SMS show social motivation associated with more negative behavioural outcomes such as self-injury and behaviours that challenge, due to high demand for individualised attention from adults [89]. In contrast with high levels of social motivation, individuals also present with clinically significant difficulties on the SCQ across domains of social-communication and RRBIs (72% [55]). Though behavioural and emotional difficulties decrease with age, social communication difficulties and repetitive behaviours persist [90]. The sex ratio commonly cited in non-syndromic autism is 4:1 and 3:1 (male:female), and this proportion reduces to 2:1 in non-syndromic ID [91]. However, there is a reversed sex difference in SMS, with three females scoring above the threshold on the SCQ per male [92•]. These sex differences are not seen for IQ, adaptive functioning, and behavioural or emotional difficulties and indicate a sex-specific pathway to behavioural autism characteristics in SMS, which may require tailored clinical support.

### Tuberous Sclerosis Complex (TSC)

Epilepsy is the most common feature of TSC and has been identified as related to autism characteristics [93•]. Seizure onset before age 1 year and greater severity of infantile spasms are positively correlated with autism characteristics [94], although the cause-effect nature of these relationships is not clear [95–97]. Up to 66% of infants with TSC meet the criteria for autism on the ADOS [98] and demonstrate a profile of social communication differences which are highly similar to that observed in non-syndromic autism [99, 100]. Social communication differences, including reduced eye contact, social babbling, and reciprocal smiling, are more frequently reported than RRBIs in infancy [101, 102]. By 36 months, early seizure onset, higher seizure frequency, and delayed language development distinguish those with TSC (+ autism) than TSC-alone [103]. As early-onset and severe epilepsy are also associated with greater severity of ID, effective treatment and prevention of epilepsy are considered vital for long-term outcomes in TSC [104].

## Key Considerations for Assessment and Support

Re-conceptualisation of autism as a ‘spectrum’ condition in the DSM-5 [105] has resulted in a single diagnosis encompassing vast behavioural heterogeneity. Furthermore, DSM criteria now state that a diagnosis of autism should only be made if social communication difficulties cannot be better explained by ID [102]. However, diagnostic guidance does not indicate when or how ID may ‘explain’’ autism-specific difficulties. A modified version on the DSM-5, the Diagnostic Manual—Intellectual Disability (DM-ID-2; 103), highlights that it can be challenging to distinguish autism from ID but does not provide guidance beyond the requirement that ‘deficits’ must exceed general delay.

Since the modification of these criteria, referrals to autism diagnostic services include a significant proportion of individuals without ID [5••]. Likewise, individuals with ID are commonly excluded from autism research [107•]. Together, this has downstream effects on clinical expertise and resources. Distinguishing autism-specific difficulties in genetic syndromes poses additional challenges. Clinicians are not only presented with the difficult task of determining when characteristics may be attributable to a person’s ID but also must consider factors associated with the behavioural phenotype and longitudinal heterogeneity of the syndrome, which are not accurately captured under classification systems. To provide valid differential diagnosis, clinicians must have sufficient understanding of not only the clinical manifestation of autism in the context of ID, but also syndrome-specific profiles of autism characteristics and co-occurring diagnoses as described above. Where there is a lack of specialism, people are likely to be misdiagnosed or precluded from access to diagnostic pathways/assessment when it is appropriate.

The validity of autism specific assessments for use within genetic syndrome populations generates a significant challenge that impacts widely across this field of research and consequently impedes clinical diagnosis. Autism assessment tools are primarily developed with non-ID populations [108, 109]. As a result, screening measures have reduced sensitivity and specificity for persons with ID, particularly for use in people with specific genetic syndromes [110••]. This can lead to an autism diagnosis being made when this is not wholly relevant to the individual and result in implementation of generic autism support which may not support individual’s needs. Importantly, this may also lead to dismissal of a diagnosis or reluctance to pursue a full autism assessment in situations where it would be appropriate and autism specific support would be beneficial. Reliance on clinical cut-off scores, which are based on a single facet of autism and have limited normative data, may mask syndrome-specific associated profiles of autism characteristics and thus compound the issue of misclassification [111]. Given this, when screening tools are used as part of a standard, clinical triage for autism assessment, a score below threshold for a person with a genetic syndrome, and ID should not be used as the sole criteria to prevent a full autism assessment. As outlined above, evidence also points towards variability within and between genetic syndrome groups regarding the emergence of autistic characteristics and related trajectories of development (e.g. 23, 24). This heterogeneity requires additional consideration regarding the timing of clinical assessment of autism in these groups.

Diagnostic observational assessments, such as the ADOS-2, have further practical limitations when used with people with ID. For instance, ADOS-2 Modules 3 and 4 require verbal fluency and measure higher-level social communication skills (e.g., reporting of event), yet verbal fluency does not always parallel cognitive ability. Modules 1 and 2 are designed for use with young children; thus, the materials and activities (e.g., playing with dolls) are not engaging or appropriate for most adults. Diagnostic algorithms are a further limitation. Authors have cautioned against interpreting scores on the ADOS-2 and ADI-R when a person has a mental age of 18–24 months [35, 112]. Fortunately, researchers have begun to explore how screening tools can be tailored and standardised in samples with ID [113] and suggested modifications to diagnostic assessment that are more appropriate for those with few and no words [114•, 115]. Though this is a step forward, an unintended consequence of focusing on ID broadly is that we are still limited in the ability to conceptualise syndrome-specific profiles of autism.

As explored above, prescriptive algorithms normed in non-syndromic populations are likely to lead to score differences which fail to represent the true nature of strengths and challenges within and across genetic syndromes. Taking a deep phenotyping approach, where an extensive battery of multiple measures is used, allows us to better understand the significance and presentation of autism characteristics in the context of specific syndromes. For instance, Roberts and colleagues [22•] accounted for cognitive abilities, adaptive functioning, anxiety, and ADHD to understand autism in pre-schoolers with FXS. This enabled them to consider differential diagnoses and establish a high degree of confidence in diagnostic determinations. As such, it is important to look beyond autism screening and diagnostic measures to understand and describe autism-related behaviours to facilitate appropriate supports. Triangulation of tools that measure social motivation/avoidance and broader *quality* of social abilities (e.g., Sociability Questionnaire for People with Intellectual Disability [43]; Child Sociability Rating Scale [114•]), restricted/repetitive behaviours (e.g., Repetitive Behaviour Questionnaire [117]) and sensory sensitivity (e.g., Sensory Experiences Questionnaire [118]) with standard autism screening and diagnostic tools will also lead to a more comprehensive picture of an individuals’ strengths and needs, and may overcome some of the limitations of the current autism screening tools. It is also important to develop an understanding of those who score below threshold for autism within genetic syndrome populations, especially where the profile of need sits within one or two of the autism diagnostic domains, but not across all domains. It is plausible that these individuals may benefit from relevant components of autism-specific support and interventions. Together, these research and clinical recommendations would support efforts to develop syndrome-sensitive algorithms and cut-off scores, which could inform operationalisation of autism diagnostic criteria in the DSM-5 (and other classification systems). We also suggest researchers develop large-scale, open-source data sets, enabling cross-syndrome comparisons both concurrently and over-time to improve the precision and replicability of autism phenotyping.

Specifically in clinical practice, scores on existing algorithms should be used in tandem with sufficient understanding of a person with a genetic syndrome. For instance, context is needed regarding their developmental level, but also differences related to the physical and behavioural phenotypes, which are likely to contribute to their overall presentation. These should be understood in relation to the developmental trajectory of autism characteristics and age-related changes associated with the syndrome—especially as older individuals are more likely to be encountered by diagnostic services, given the reported delays in access to assessment [6, 7••]. Assessment of potential co-occurring conditions (e.g., ADHD, social anxiety) would also support differential diagnosis. If co-occurring conditions are present, then it is important to consider how it may influence the validity of autism diagnostic assessment and, if possible, consult relevant professionals.

Though much of this review has focused on differential diagnosis, we finish by emphasising the urgent need for change in service provision to value need over diagnosis. People with genetic syndromes who present with ‘atypical’ profiles of autism characteristics may still benefit from clinical and educational support strategies primarily developed for autistic people who do not have a genetic syndrome. Depending on the country, health service provision, and national guidelines, clinical services differ in terms of eligibility criteria and funding. Yet it is often the case that autism services are perceived as being more comprehensive than those available for other neurodevelopmental conditions [119] but also somewhat disconnected from other diagnostic and disability services. Given the high rates of co-occurrence across neurodevelopmental conditions, greater convergence of clinical services and support would be beneficial [120••]. Understandably, these practical factors may motivate caregivers and professionals working with an individual to seek a diagnosis to support access to such provisions. Amassing an evidence base for differential diagnosis in each syndrome is an ambitious goal, and if services wait for this goal to be achieved, immediate support for people with these syndromes will be precluded or significantly delayed. As such, researchers argue that a needs-led approach may be the better alternative to a categorical diagnosis [5••]. Where it is possible and appropriate to translate findings from the non-syndromic autism literature to people with genetic syndromes, clinical services should seek to do so, to accelerate the progress of practice-based evidence and improve real-world outcomes and support for people with genetic syndromes.

## Conclusions

The behavioural and longitudinal heterogeneity of autism-related behaviours within and across genetic syndromes indicate some degree of syndrome specificity, as illustrated in the examples provided above. Current classification systems and diagnostic assessment tools do not provide clear guidance on how to disentangle these differences from associated ID and broader phenotypic characteristics. Therefore, differential diagnosis relies on the development of syndrome-sensitive assessment practices, alongside access to comprehensive clinical expertise, to establish strengths and challenges as a baseline. However, access to support should not be dependent on diagnostic categorisation. Autism characteristics in genetic syndromes demand attention across time and circumstance, to evidence and support related changes in need.

## Footnotes

1 In most cases, autism is diagnosed in people who do not have a known genetic syndrome. In this paper, we have used the term ‘non-syndromic autism’ to reflect such cases.

2 The term autism has been chosen over the diagnostic term autism spectrum disorder (ASD) wherever possible to reflect the view that autism is a difference rather than a dysfunction [137]). This is consistent with the neurodiversity perspective [138] and the deficit-as-difference conception of autism [139]. However, where essential to maintain precision in reporting, the diagnostic term ‘ASD’ is used. The identity-first phrasing ‘autistic people’ is also used, as it is the preferred term by the UK autism community [140].

3 ‘ + autism’ is used to distinguish people within a genetic syndrome group who have received a clinical diagnosis of autism.
